# Efficacy of intraoperative fluorescence imaging using indocyanine green‐containing gauze in identifying the appropriate dissection layer in laparoscopic intersphincteric resection: A case report

**DOI:** 10.1002/ccr3.6356

**Published:** 2022-10-20

**Authors:** Hiroyuki Kumata, Keisuke Onishi, Tetsuro Takayama, Kengo Asami, Noriyuki Obara, Hirofumi Sugawara, Izumi Haga

**Affiliations:** ^1^ Department of Surgery Japan Community Health Care Organization Sendai Hospital Sendai Miyagi Japan; ^2^ Department of Surgery Yamagata City Hospital Saiseikan Yamagata Yamagata Japan; ^3^ Department of Gastroenterology Japan Community Health Care Organization Sendai Hospital Sendai Miyagi Japan

**Keywords:** indocyanine green, intraoperative fluorescence imaging, laparoscopic intersphincteric resection

## Abstract

In laparoscopic intersphincteric resection, identifying the dissection layer near the anus is often difficult. We safely proceeded with it, using indocyanine green‐containing gauze on the anal side to remove the internal anal sphincter with indocyanine green fluorography.

## 1 INTRODUCTION

Intraoperative indocyanine green (ICG) fluorography can be helpful for various digestive surgeries^1^ and has a wide range of uses, such as in the evaluation of blood or bile flow, lesion localization, and boundary between the lesion and normal tissue.^2^, ^3^, ^4^, ^5^ ICG is generally injected into the patient's body via the intravenous or subcutaneous routes.

Determining the exact dissection layer between the internal and external anal sphincters for laparoscopic intersphincteric resection (ISR) is often difficult.^6^ Furthermore, matching the approached dissection layer from the transanal surgery with that from the transpelvic surgery is quite challenging. Here, we proposed a procedure that can help alleviate the difficulty in identifying a suitable dissociated layer by impregnating gauze with ICG without injecting it into the patient's body during laparoscopic ISR.

## 2 CASE HISTORY

**FIGURE 1:**
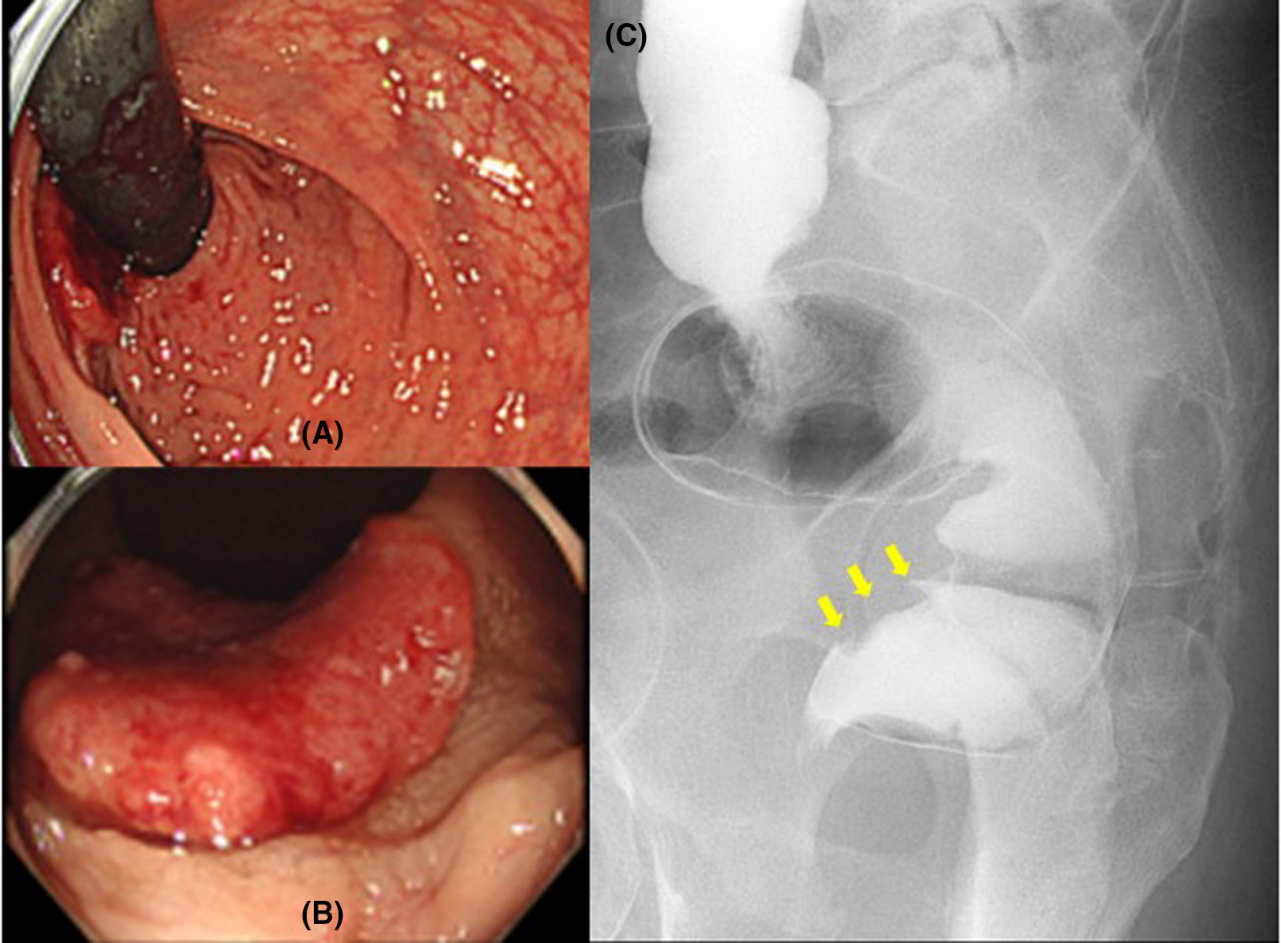
Preoperative findings. (A, B) Colonoscopy showing a mass lesion approximately 2 cm in diameter at the lower rectum (A: Looking up image, B: Front image). The degree of lesion invasion on endoscopic evaluation suggested infiltration into the deep submucosal layer. (C) Radiographic contrast enema image showing that the lower edge of the lesion was approximately 3 cm away from the dentate line (the lesion is in the area of the yellow arrows indicating the location of the lesion).

**FIGURE 2:**
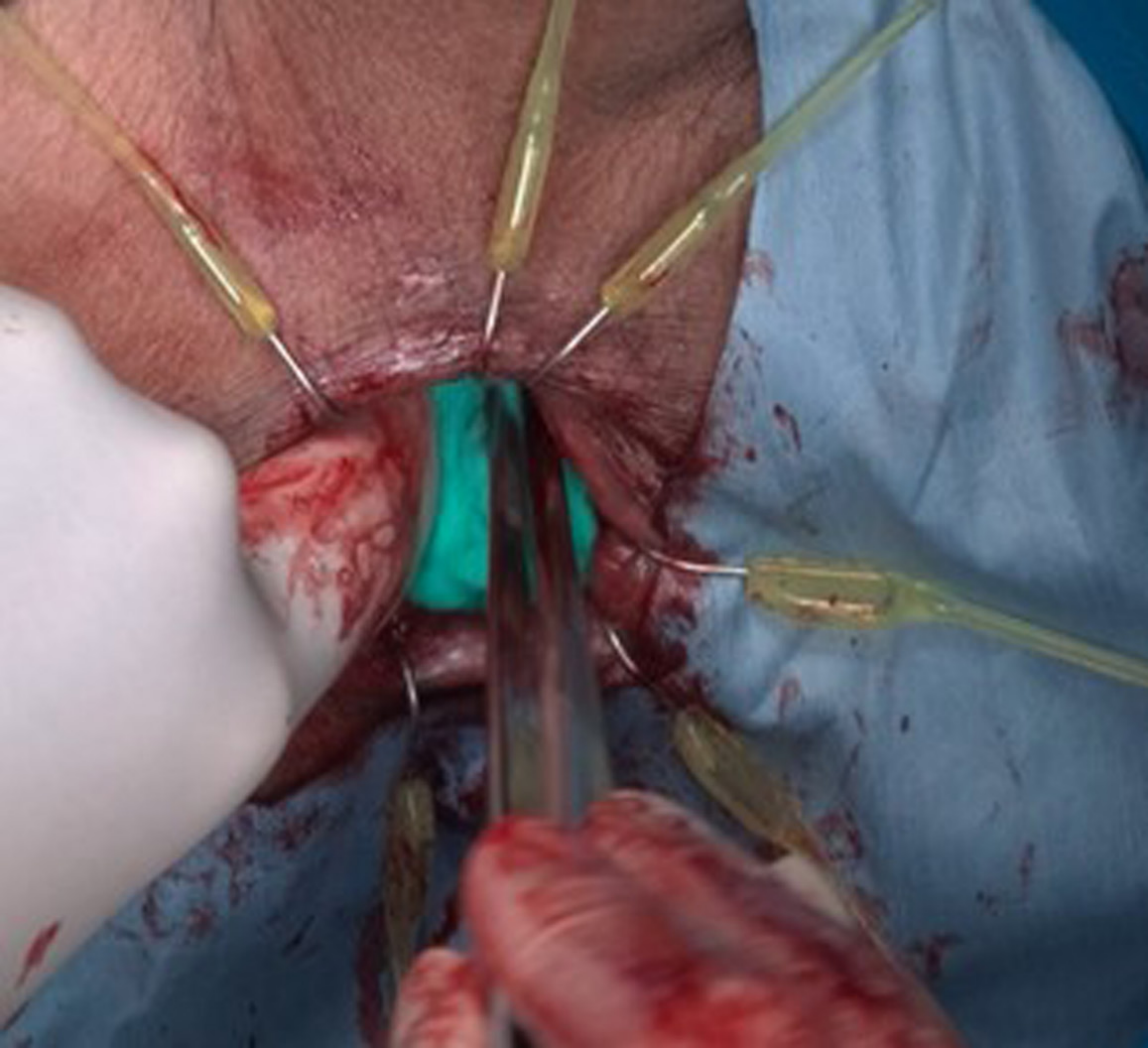
Surgical findings of the transanal procedure. We entered the layer between the internal and external sphincters and proceeded with the detachment of the pelvic muscles to the extent that the prostate was palpable on the ventral side and the coccyx on the dorsal side. After peeling to that depth, we spread three pieces of gauze all around the deepest parts. The gauze contained a liquid prepared by dissolving 25 mg of ICG in 10 ml of water containing sodium arginine.

**FIGURE 3:**
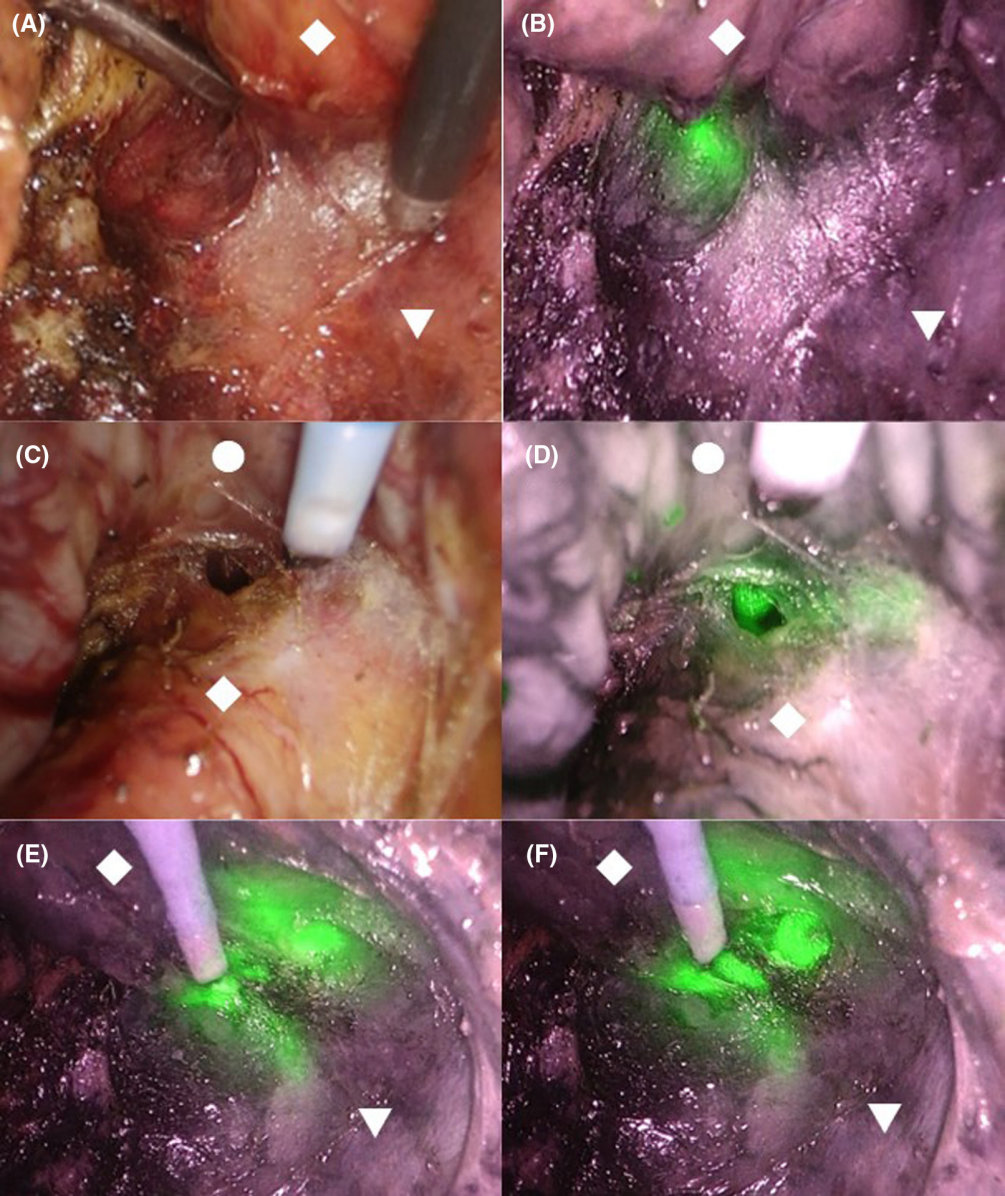
Surgical findings of laparoscopic manipulation. In the infrared observation mode, the luminescent gauze with indocyanine green was seen through the back of the tissue (A: normal mode. B: infrared observation mode). It can be seen that the gauze was exposed by switching to the near‐infrared mode, even if it was not clear in the normal mode that the layer of the transanal approach was reached (C: normal mode. D: infrared observation mode). The target separation line can be safely reached by cutting the membrane at a site with strong luminescence (E, F). As the fibrous tissue was exfoliated, the fluorescent gauze gradually became see‐through (E). When the tissue was further incised with an electric knife at the site where the fluorescence was strong, the gauze was finally exposed to the naked eye (F). ◆: Rectum. ▼: Levator ani muscle. ●: Prostate.

A 64‐year‐old man with no medical history received a positive stool test for fecal occult blood. The patient showed no notable abnormal findings on upper gastrointestinal endoscopy. He subsequently underwent colonoscopy and was found to have a mass lesion approximately 2 cm in diameter at the lower rectum (Figure 1). Endoscopic evaluation of lesion invasion suggested infiltration into the deep submucosal layer. A biopsy revealed a histopathological diagnosis of moderately differentiated tubular adenocarcinoma. Rectal examination of the lesion showed that the lower edge of the lesion was about 3 cm away from the dentate line and had good mobility (Figure 1). No other gross lesions were noted after examining the entire large intestine. No notable abnormal findings were observed in terms of general biochemistry, complete blood count, or coagulation test. Tumor markers, including carcinoembryonic antigen and carbohydrate antigen 19‐9 levels, were also within normal ranges. Computed tomography showed no clear localization of the rectal mass and no significant lymphadenopathy around the lower rectum. None of the findings suggested obvious distant metastasis. Based on the mentioned findings, we determined that surgical resection was indicated for this lesion. Furthermore, based on the positional relationship between the mass and the dentate line, we determined that ISR was appropriate and decided to perform laparoscopic ISR considering that the patient had no history of laparotomy. The patient was placed in the lithotomy position intraoperatively with his head low. We started the surgery via the transanal procedure. Accordingly, we applied the Lone Star RETRACTOR™ all around the anal canal and observed the inside of the rectum. As shown in the preoperative examination findings, the dentate line was 3 cm away from the lower edge of the mass. We dissected all layers of the rectum with a 2 cm margin from the inferior margin of the mass to the anal side. We closed the rectal stump on the resected side with a nodular suture, after which we entered the layer between the internal and external sphincters and proceeded with the detachment of the pelvic muscles to the extent that the prostate was palpable on the ventral side and the coccyx on the dorsal side. After peeling to that depth, we prepared three pieces of 10 cm square small gauze, which was soaked in a liquid prepared by dissolving 25 mg of ICG in 10 ml of water containing sodium arginine with a concentration of 6 mg/ml for approximately 30 s, and then sufficiently squeezed out as much as possible until completely dry, and carefully spread it all around the deepest parts so that they were in the same layer (Figure 2). Thereafter, we proceeded with laparoscopic manipulation starting with five ports. We proceeded with detachment from the inside of the sigmoid mesentery and dissected the lymph nodes around the root of the inferior mesenteric artery (IMA). We then dissected the IMA at the height immediately after the left colic artery diverged and then performed rectal and mesenteric detachment. As we peeled the rectum from the front of the sacrum and proceeded toward the anus, we encountered the levator ani muscle. After switching the scope to the near‐infrared observation mode while further peeling toward the anus and paying attention to the levator ani muscle, the luminescent gauze was observed through the back of the tissue. As the fibrous tissue was exfoliated, the fluorescent gauze gradually became see‐through. When the tissue was further incised with an electric knife at the site where the fluorescence was strong, the gauze was finally exposed to the naked eye. We then proceeded with rectal detachment to expose the entirety of gauze while relying on fluorescent ICG and paying attention to the prostate, seminal vesicles, and neurovascular bundle, which allowed us to safely reach the detached layer from the anal side (Figure 3). Thereafter, we returned to the transanal surgery once more. The intestinal tract was dragged out of the anus and the sigmoid colon was dissected to remove the specimen. Afterward hat, the sigmoid colon and the residual rectum were sutured with a total of 16 needles around the circumference with a 4‐0 monofilament absorbent thread in all layers and anastomosed. We then confirmed the absence of intra‐abdominal bleeding via laparoscopy, after which a temporary ileal‐covering stoma was finally constructed and the surgery was completed. The operative time and blood loss were 293 min and 5 ml, respectively.

## 3 OUTCOME AND FOLLOW‐UP

The postoperative course was good, and the patient was discharged on the 16th postoperative day with a final histopathologic diagnosis of Stage I (pT2, N0 [0/12], M0) carcinoma of lower rectum, margins of resection clear. One month after the surgery, a recto‐anal pressure test and rectal‐anal sensation test were performed, the results of which were similar to those before the surgery, suggesting no problems. No obvious recurrence was observed 6 months after the surgery.

## 4 DISCUSSION

In recent years, ICG‐guided surgery has gained popularity in the field of gastrointestinal surgery^4^ given that it allows for enhanced real‐time intraoperative visualization of anatomical structures, vascular perfusion, and lymph node navigation. To achieve the mentioned purposes, ICG has generally been administered intravenously. During gastrointestinal surgery, the intraoperative ICG navigation system is often used mainly for evaluating blood flow at the distal end of the gastrointestinal tract.^5^ Intraoperative ICG fluorescence imaging has been proven very useful for evaluating blood flow at the anastomotic site even during laparoscopic surgery for colorectal cancer, especially rectal cancer, and contributes toward reducing the risk of anastomotic leakage.^5^ During rectal cancer surgery, performing the dissection procedure at the appropriate pelvic layer is just as important as the evaluation of the anastomotic site condition.^7^ Appropriate nerve preservation with maximum consideration for lymph dissection is of course important; however, proper dissection of the pelvic floor muscles to preserve defecation function is also critical for future patient quality of life.^8^ In particular, surgery to preserve the anus of patients with lower rectal cancer requires preserving the appropriate pelvic floor muscles, with an emphasis on defecation function and curative surgery. ISR is the ultimate sphincter‐preserving surgery for very low rectal cancers.^7^ We discussed how the ISR can help secure a proper dissection line and preserve the external anal sphincter. After examining ISR cases thus far, we found that it was important to smoothly connect the dissociated layer from the transanal approach to that from the transperitoneal approach.^9^ We believed that this problem could be solved by visualizing the dissected layer of the transanal approach from the abdominal cavity side and navigating via ICG intraoperative fluorescence imaging. A problem with this procedure is that if the water content of the gauze is not squeezed out, the gauze will retain a large amount of liquid and the ICG will seep into the surrounding tissue, making it impossible to achieve pinpoint luminescence from the exfoliation layer, which makes distinguishing the dissociation layer challenging. To address this problem, we mixed ICG with sodium arginine to make it viscous and then forcefully squeezed the gauze as much as possible to eliminate excess moisture. With this approach, the ICG could be visualized transabdominally without exuding into the surroundings. Besides, if gauze is placed in the wrong layer in the transanal approach, it may be difficult to determine the appropriate layer during laparoscopic operation. To the best of our knowledge, no reports have used ICG intraoperatively in this manner. Another merit of using this approach for ICG is that it is not administered intravenously, which potentially reduces allergic reactions and the burden on the patient's body. Confirmation of the dissected layer via intraoperative fluorescence imaging with a gauze containing ICG seems to be applicable to other types of cancers aside from rectal cancer and may also be suitable for obese patients in whom identifying the dissected layer may be a difficulty.

We herein reported a unique and advantageous method of using ICG during surgery. Nonetheless, more similar cases should be accumulated in order to establish a consensus on the treatment strategy.

## AUTHOR CONTRIBUTIONS

Hiroyuki Kuamata, Keisuke Onishi, and Tetsuro Takayama performed the surgery. Hiroyuki Kumata, Tetsuro Takayama, Kengo Asami, and Izumi Haga managed the perioperative course. Noriyuki Obara was the gastroenterologist who examined and diagnosed the cancer. Hiroyuki Kumata, Keisuke Onishi, Tetsuro Takayama, Kengo Asami, Hirofumi Sugawara, and Izumi Haga participated in discussions. Keisuke Onishi supervised the patient treatment. Keisuke Onishi and Tetsuro Takayama prepared the manuscript. Kengo Asami supervised the writing of the manuscript. All authors have read and approved the final manuscript.

## CONFLICT OF INTEREST

The authors declare that they have no conflict of interest.

## ETHICS STATEMENT

All procedures followed were in accordance with the ethical standards of the relevant committee on human experimentation (institutional and national) and with the Helsinki Declaration of 1975, as revised in 2008.5

## CONSENT

Written informed consent was obtained from the patient to publish this report in accordance with the journal's patient consent policy.

## Data Availability

The data that support the findings of this study are openly available in [repository name e.g “figshare”] at http://doi.org/10.1002/ccr3.6356, reference number [^9^].
